# Plasmin Plays an Essential Role in Amplification of Psoriasiform Skin Inflammation in Mice

**DOI:** 10.1371/journal.pone.0016483

**Published:** 2011-02-02

**Authors:** Qun Li, Fang Ke, Weiwei Zhang, Xiaoyan Shen, Qiannan Xu, Hong Wang, Xue-Zhong Yu, Qibin Leng, Honglin Wang

**Affiliations:** 1 Shanghai Institute of Immunology, Institute of Medical Sciences, Shanghai Jiao Tong University School of Medicine, Shanghai, China; 2 Vascular Biology Laboratory of Department of Hypertension of Shanghai Ruijin Hospital, Shanghai Institute of Hypertension and Institute of Health Sciences, Shanghai Jiao Tong University School of Medicine and Shanghai Institutes for Biological Sciences, Chinese Academy of Sciences, Shanghai, China; 3 Department of Dermatology, Ruijin Hospital, Shanghai Jiao Tong University School of Medicine, Shanghai, China; 4 H. Lee Moffitt Cancer Center & Research Institute, Tampa, Florida, United States of America; 5 Institut Pasteur of Shanghai, Chinese Academy of Sciences, Shanghai, China; Agency for Science, Technology and Research (A*STAR), Singapore

## Abstract

**Background:**

Although increased levels of plasminogen activators have been found in psoriatic lesions, the role of plasmin converted from plasminogen by plasminogen activators in pathogenesis of psoriasis has not been investigated.

**Methodology/Principal Findings:**

Here we examined the contribution of plasmin to amplification of inflammation in patients with psoriasis. We found that plasminogen was diminished, but that the amount and activity of its converted product plasmin were markedly increased in psoriasis. Moreover, annexin II, a receptor for plasmin was dramatically increased in both dermis and epidermis in psoriasis. Plasmin at sites of inflammation was pro-inflammatory, eliciting production of inflammatory factors, including CC chemokine ligand 20 (CCL20) and interleukin-23 (IL-23), that was mediated by the nuclear factor-kappaB (NF-κB) signaling pathway and that had an essential role in the recruitment and activation of pathogenic C-C chemokine receptor type 6 (CCR6)^+^ T cells. Moreover, intradermal injection of plasmin or plasmin together with recombinant monocyte/macrophage chemotactic protein-1 (MCP-1) resulted in induction of psoriasiform skin inflammation around the injection sites with several aspects of human psoriasis in mice.

**Conclusions/Significance:**

Plasmin converted from plasminogen by plasminogen activators plays an essential role in amplification of psoriasiform skin inflammation in mice, and targeting plasmin receptor - annexin II - may harbor therapeutic potential for the treatment of human psoriasis.

## Introduction

Psoriasis is a chronic skin disorder characterized by a dense dermal infiltrate, which predominantly consists of T cells, dendritic cells (DCs), natural killer T cells, and macrophages, and affects approximately 0.2% to 2% of the world population [1]. Plasminogen (PLG) is mainly produced in the liver and is activated by PLG activators, other serine proteases [2], or streptokinase (SK) secreted by group A streptococci (GAS) [3]. PLG activators are highly elevated in the lesional skin of psoriasis patients, suggesting that PLG is involved in the pathogenesis of psoriasis [4], [5], [6]. Moreover, uninvolved skin from patients with psoriasis develops signs of psoriatic lesions accompanied with increases in PLG activator activity after being grafted on nude mice [7]. The role of plasmin (PL) converted from PLG in inflammation has been widely studied, and apart from its fibrinolytic functions, PL is a potent cell activator [8]. However, no direct evidence for the contribution of PL to the pathogenesis of psoriasis has been reported.

An initial cellular event in the development of psoriatic lesions is the infiltration of target skin by activated pathogenic T cells, including Th17 T cells. These activated T cells produce inflammatory mediators, such as IFN-γ, IL-17 and IL-22, induce epidermal hyperplasia and over-expression of key molecules, which may act with keratinocytes, DCs and dermal macrophages to sustain a cycle of inflammation leading to the psoriatic phenotype [9], [10], [11], [12]. In psoriasis, CCL20 produced at sites of skin inflammation is a chemoattractant for skin-homing cutaneous lymphocyte-associated antigen (CLA)^+^CCR6^+^ T cells and Th17 cells in the circulation [13], [14]. A critical role for CCR6 in the pathogenesis of psoriasis has been recently demonstrated [15]. In addition, treating cultured human keratinocytes with IL-17 was shown to cause dose- and time-dependent up-regulation of CCL20, leading to the speculation that inhibition of this process may contribute to the resolution of psoriasis [16].

Similarly, IL-23, which is a novel cytokine important for autoimmunity, consisting of an IL-23 p19 chain and an IL-23 p40 chain, is regulated by several transcription factors including AP-1 and NF-κB [17]. IL-23 is now recognized to play an essential role in the activation of inflammatory cells in Th1-mediated diseases including psoriasis, where it is expressed in monocytes and dendritic cells of lesional human skin [18]. Intradermal administration of IL-23 into murine skin initiates a cascade of events including erythema, mixed dermal infiltrates, and epidermal hyperplasia via induction of the NF-κB-dependent TNF [19]. Furthermore, a recent genome-wide analysis for psoriasis susceptibility loci revealed an association of psoriasis with IL-12, IL-23 and NF-κB-dependent pathways [20], stressing the potential value of our recent approach by targeting NF-κB activation for the treatment of psoriasis [21].

Despite the substantial progress in understanding the molecular mechanisms underlying psoriasis, the direct evidence of the contribution of PL to pathogenesis of psoriasis is still lacking. In this study, we show that PLG is diminished, but that the amount and activity of PL are markedly increased in psoriasis. The role of PL at sites of inflammation was pro-inflammatory, eliciting production of inflammatory factors, including CCL20 and IL-23, that was mediated by the NF-κB signalling pathway and that had an essential role in the recruitment and activation of pathogenic CCR6^+^ T cells. Strikingly, intradermal injection of PL or PL together with recombinant MCP-1 resulted in induction of psoriasiform skin inflammation around the injection sites with several aspects of human psoriasis in mice. Thus, our findings for the first time provide a potential mechanism for how PL converted from PLG by PLG activators is involved in initiation and/or worsening psoriasis by inducing the recruitment and activation of skin-homing pathogenic T cells or by directly activating keratinocytes in patients, suggesting that targeting PL receptor - annexin II - may harbor therapeutic possibility for the treatment of psoriasis.

## Results

### Expression of PLG is significantly diminished in psoriasis

**Figure 1:**
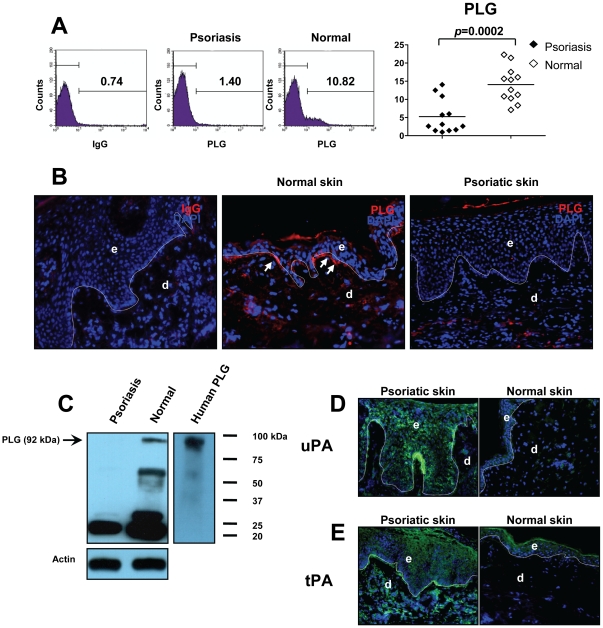
Diminished PLG in psoriasis. (**A**) A significant reduction in the expression of PLG in PBMC of psoriasis patients was detected by flow cytometry. (**B**) Cryosections from lesional skin of psoriasis patients or normal volunteers were stained with a PLG mAb (red). (**C**) Lysates from lesional skin of psoriasis patients or normal skin of volunteers were subjected to western blotting for PLG expression. Human PLG was used as a positive control (right panel). Actin - loading control. (**D, E**) Cryosections from lesional skin of psoriasis patients or normal volunteers were stained with uPA or tPA mAbs (green) for its expression. Original magnification for immunofluorescent staining, ×400. e, epidermis; d, dermis. Dotted lines indicate the border between epidermis and dermis.

Both PLG activators and streptococcus-derived SK contribute to the conversion of PLG to PL [3]. Because cell-bound PLG is more efficiently activated to PL, we first examined the expression of PLG in PBMC from patients with psoriasis by flow cytometry [22]. Staining with a specific mAb against human PLG demonstrated that cell-bound PLG was significantly decreased in PBMC from patients compared with normal individuals (14.07±4.85 vs. 5.24±4.69, *p* = 0.0002, n = 12) (**Figure 1A**). Immunofluorescent staining of biopsies from lesional skin of patients with chronic plaque-type psoriasis demonstrated that PLG was diminished in psoriatic skin (**Figure 1B** **right**). In contrast, in normal skin derived from healthy individuals, a clear staining for PLG was observed, particularly in the basal layer of epidermis (**Figure 1C** **middle, white arrows**). Western blot data further revealed decreased expression of PLG in psoriatic skin compared to normal skin from healthy donors (**Figure 1C**). PLG has been suggested to be involved in the pathology of psoriatic lesions because lesional psoriatic epidermis has increased levels of PLG activators, known as urokinase PA (uPA) and tissue PA (tPA), compared to the epidermis of uninvolved skin and normal skin [7], [23]. Consistent with this report [7], we detected a very strong staining of uPA on epidermal keratinocytes of psoriatic lesions compared with normal controls (**Figure 1D**). Furthermore, in contrast to normal skin we have found that tPA was expressed by both epidermal keratinocytes and dermal cells in psoriatic lesions (**Figure 1E**). Collectively, our data implied that intact, cell-bound PLG was diminished in psoriasis, and this decrease may due to increased expression of its activators, uPA and tPA.

### PL activity is enhanced in psoriasis

**Figure 2:**
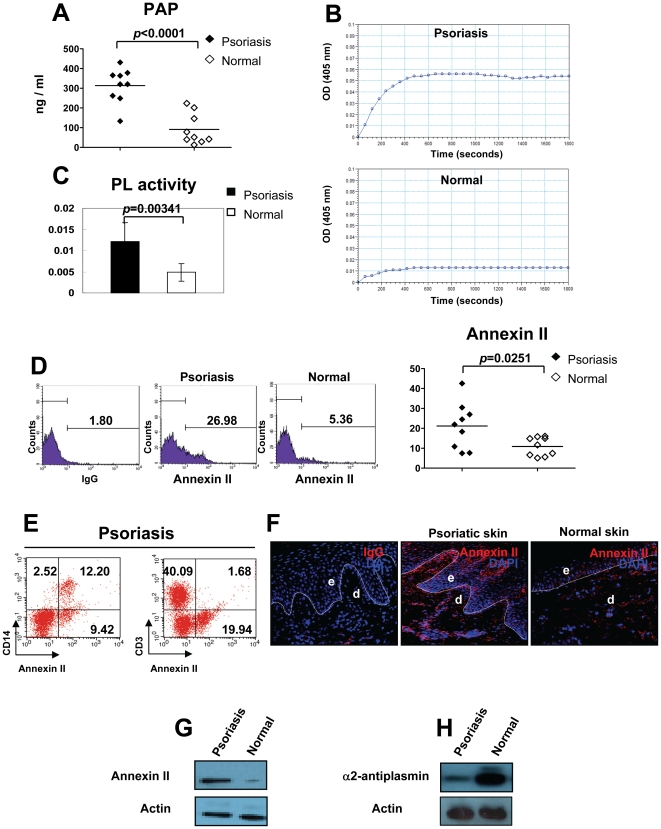
Enhanced PL activity in psoriasis. (**A**) PL was measured by ELISA. (**B**) PL activity was detected by a chromogenic assay. (**C**) The PL activity was represented by ΔA/min (absorbance/minute). (**D**) The percentage of annexin II^+^ cells in PBMC of psoriasis patients and normal controls is shown. (**E**) Expression levels of annexin II on CD14^+^ monocytes and CD3^+^ T cells in PBMC of psoriasis patients was measured by Flow cytometry. One representative experiment out of 5 is shown. (**F**) Cryosections from lesional skin of psoriasis patients or normal volunteers were stained with an annexin II mAb (red). Original magnification, ×400. e, epidermis; d, dermis. Dotted lines indicate the border between epidermis and dermis. (**G, H**) Lysates from lesional skin of psoriasis patients or normal skin of volunteers were subjected to western blotting for annexin II expression or α2-antiplasmin expression. Actin - loading control.

Production of PL can be examined by measuring the circulating plasmin-α2 antiplasmin complexes (PAP). We next measured the total amount of PAP in plasma by ELISA. PAP levels were significantly elevated in patients with psoriasis compared with controls (313.71±21.49 vs. 91.51±26.24, *p*<0.0001, n = 9) (**Figure 2A**). Using a chromogenic assay, we assessed activity of PL in psoriasis. We found that levels of PL activity in plasma derived from psoriasis patients were markedly increased compared with normal volunteers (**Figure 2B**), and this difference between patients and controls is statistically significant (0.0122±0.0043 vs. 0.0048±0.0021, *p* = 0.0034, n = 9) (**Figure 2C**). Annexin II is a receptor for PL, and binding of PLG to annexin II on the cell surface markedly enhances production of PL [24]. We observed that there was a significant increase in cell surface expression of annexin II on PBMC derived from psoriasis patients than on PBMC from controls (21.20±11.59 vs. 10.90±4.68, *p* = 0.0251, n = 9) (**Figure 2D**). Furthermore, we found that CD14^+^ monocytes, but not CD3^+^ T cells predominantly expressed annexin II in PBMC of patients with psoriasis (**Figure 2E** **)**. Immunofluorescent staining of biopsies from psoriatic lesions demonstrated that annexin II was dramatically increased in both dermis and epidermis (**Figure 2F** **middle**), whereas in normal skin derived from controls, only a weak staining for annexin II was found (**Figure 2F** **right**). Western blot further confirmed an enhanced expression of annexin II in psoriatic skin compared to normal skin from healthy donors (**Figure 2G**). α2-antiplasmin contributes to the inactivation of PL activity. Strikingly, our western blot data revealed a prominent decrease in the expression of α2-antiplasmin in psoriatic lesions (**Figure 2H**). These results constitute evidence that the activity of PL is excessive in patients with psoriasis, and the persistence of PL activity may be favored by increased expression of annexin II and insufficient production of α2-antiplasmin.

### PL induces psoriasiform inflammation in mouse ear skin

**Figure 3:**
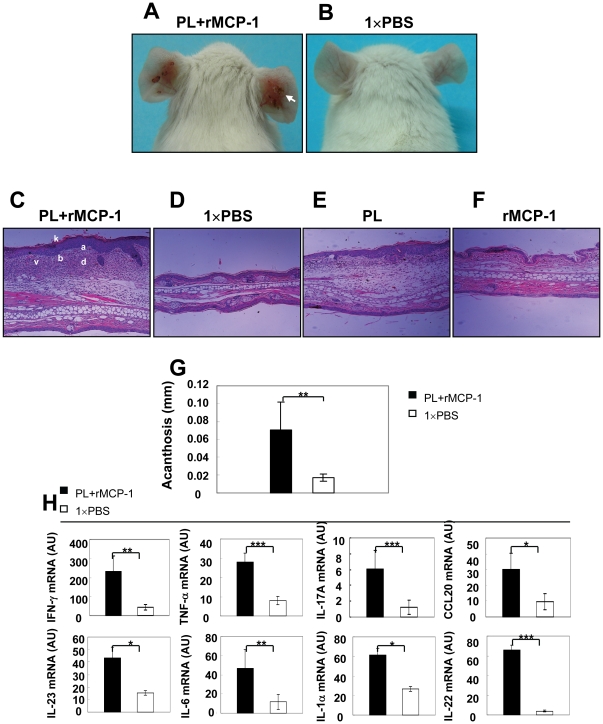
Psoriasiform skin disease induced by PL in mice. The ears of WT BALB/c mice were injected intradermally every other day for 28 days with 30 µl 1×PBS either containing 1.5 µg PL and 30 ng recombinant murine MCP-1 or alone. (**A, B**) Representative clinical features of ears of treated mice. White arrow indicates severe erythema, scales and crusts. (**C–F**) H&E staining of ears of from mice treated with PL+rMCP-1, 1×PBS, PL and rMCP-1. k, hyperparatosis with intracorneal neutrophilic pustule; a, acanthosis; b, increased proliferative basal layer epidermal keratinocytes; d, dermal cell infiltrate; v, dilated dermal blood vessels. Original magnification, ×100. (**E**) Microscopic changes of ears quantified using acanthosis. (**F**) Real-time RT-PCR analysis of ears with indicated treatments. * *p*<0.05, ** *p*<0.01, *** *p*<0.001, Student's *t* test.

Previously, we have shown that administration of murine recombinant TNF-α (rTNF-α) together with murine recombinant MCP-1 (rMCP-1) locally induced TNF-α release and psoriasiform skin lesions in mice [11]. To investigate whether the synergistic actions of rMCP-1 as a recruitment factor for macrophages and PL as a macrophage-activating protein may result in psoriasiform skin inflammation, we simultaneously injected PL and rMCP-1 in mouse ear skin. Indeed, 28 days after administration of PL together with rMCP-1 in healthy ears, we were able to induce progressive skin lesions around the injection site that were characterized by severe erythema, scale and crust formation (**Figure 3A** **, white arrow**). In contrast, control mice did not show any pathological skin phenotype (**Figure 3B**). Histological analysis of skin sections from mice treated with PL and rMCP-1 revealed acanthosis, hyperorthokeratosis, subcorneal microabscesses, dilated lymphatic vessels and a diffuse inflammatory infiltrates in the dermis (**Figure 3C**), compared to normal epidermis and dermis in the ears of control mice (**Figure 3D**). Additionally, we found that injection of PL alone, but not rMCP-1, could also induce abnormal proliferation of epidermal keratinocytes, however with less severity of inflammation compared to combined injection of PL and rMCP-1 (**Figure 3E, F**). Further analysis of the ear skin of mice treated with PL and rMCP-1 demonstrated a significant increase of acanthosis when compared with controls (**Figure 3G**). Moreover, real-time RT-PCR analysis was carried out on RNA isolated from the ears of wild type (WT) BALB/c mice six hours post-injection of PL and rMCP-1. We demonstrated that psoriasis-related transcripts, such as IFN-γ, TNF-α, IL-17A, CCL20, IL-23, IL-6, IL-1α and IL-22, were significantly enhanced in mouse ears treated with PL and rMCP-1 than those with 1×PBS control (**Figure 3H**). Our data suggest the proteolytic enzyme PL may have a critical role in the progress of psoriasis.

### PL-induced psoriasiform skin changes require CCR6^+^ T cells

**Figure 4:**
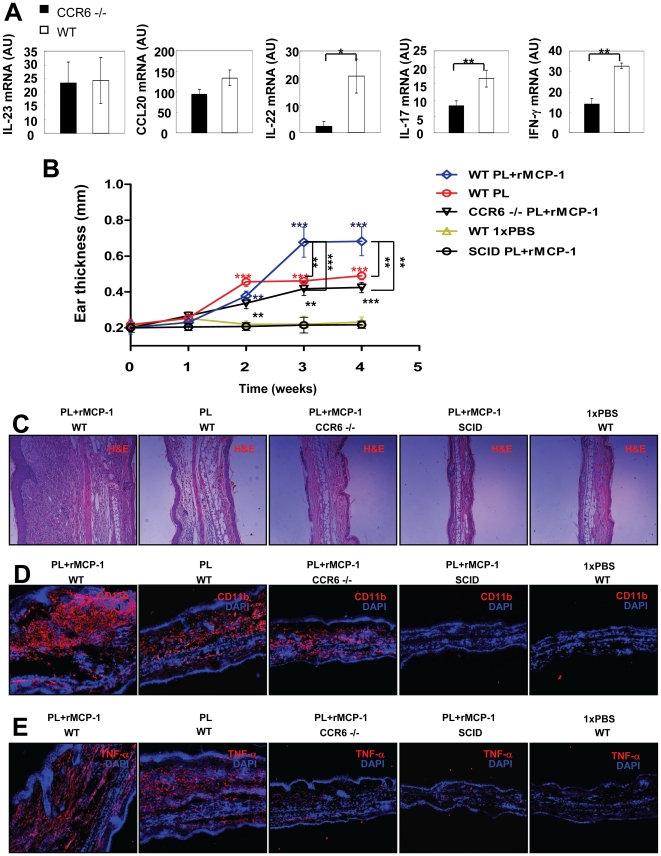
Requirement for CCR6^**+**^ T cells in the development of PL-induced psoriasiform skin changes. The ears of CCR6−/− and WT C57BL/6J mice were injected intradermally with different treatment. (**A**) Real-time PCR analysis of IL-23, CCL20, IL-22, IL-17A and IFN-γ transcripts six hours after injection. (**B**) Ear thickness of mice with indicated genotypes or treatments was measured before and at multiple time points after injection. (**C**) On day 28, ears were collected for H&E staining. Original magnification, ×100. (**D, E**) On day 28, cryosections from ears were stained with CD11b (red) and TNF-α (red). Original magnification, ×100. * *p*<0.05, ** *p*<0.01, Student's *t* test.

CCR6 has been reported to be crucial in the pathogenesis of psoriasis [15]. To identify the role of CCR6 in the development of psoriasiform skin inflammation induced by PL, we injected PL and rMCP-1 in the ears of CCR6 knockout (CCR6−/−) mice. Six hours after intradermal injection of PL and rMCP-1, we found no significant difference in IL-23 and CCL20 mRNA expression between CCR6−/− mice and WT mice (**Figure 4A**). Remarkably, expression levels of IL-22, IL-17A and IFN-γ were significantly decreased in CCR6−/− mice compared to WT mice (**Figure 4A**). These data imply that PL can induce the release of IL-23 and CCL20 independent of CCR6^+^ T cells but that, in contrast, the production of IL-22, IL-17A and IFN-γ requires CCR6^+^ T cells. Next, we injected PL or PL and rMCP-1 or 1×PBS in the ears of WT, CCR6−/− and SCID mice every other day for a period of 28 days. Both WT and CCR6−/− ears exhibited marked swelling starting two weeks after the initial injection of PL or combination of PL and rMCP-1 compared with SCID and WT control mice injected with 1×PBS (**Figure 4B**), indicating that PL was capable of directly inducing keratinocyte proliferation without the infiltration of CCR6^+^ T cells in the dermis. However, our data demonstrated a less pronounced ear thickness in WT mice injected with PL alone or in CCR6−/− mice injected with PL and rMCP-1 when compared to WT mice with the same treatment (**Figure 4B**). H&E staining of sections from ears injected with PL or PL and rMCP-1 showed acanthosis, dermal inflammation, and dilation of dermal vasculature in WT mice (**Figure 4C**). In contrast, a minor effect of PL and rMCP-1 injection was observed in CCR6−/− mice, and almost no effect of PL and rMCP-1 injection was observed in SCID mice (**Figure 4C**). Immunofluorescent staining of biopsies from ears injected with PL and rMCP-1 revealed high infiltration of CD11b^+^ macrophages in the ear skin of both WT and CCR6−/− mice, but not in that of SCID mice in comparison with controls (**Figure 4D**), suggesting that in this experimental setting, recruitment of CD11b^+^ macrophages was not blocked by the lack of CCR6. Strikingly, we detected a remarkably decreased expression of TNF-α in CCR6−/− ears injected with PL and rMCP-1 compared with WT mice (**Figure 4E**), indicating that CCR6^+^ T cells are indispensable for the production of TNF-α and for full development of psoriasiform skin changes.

### PL triggers NF-κB-dependent IL-23 and CCL20 expression

**Figure 5:**
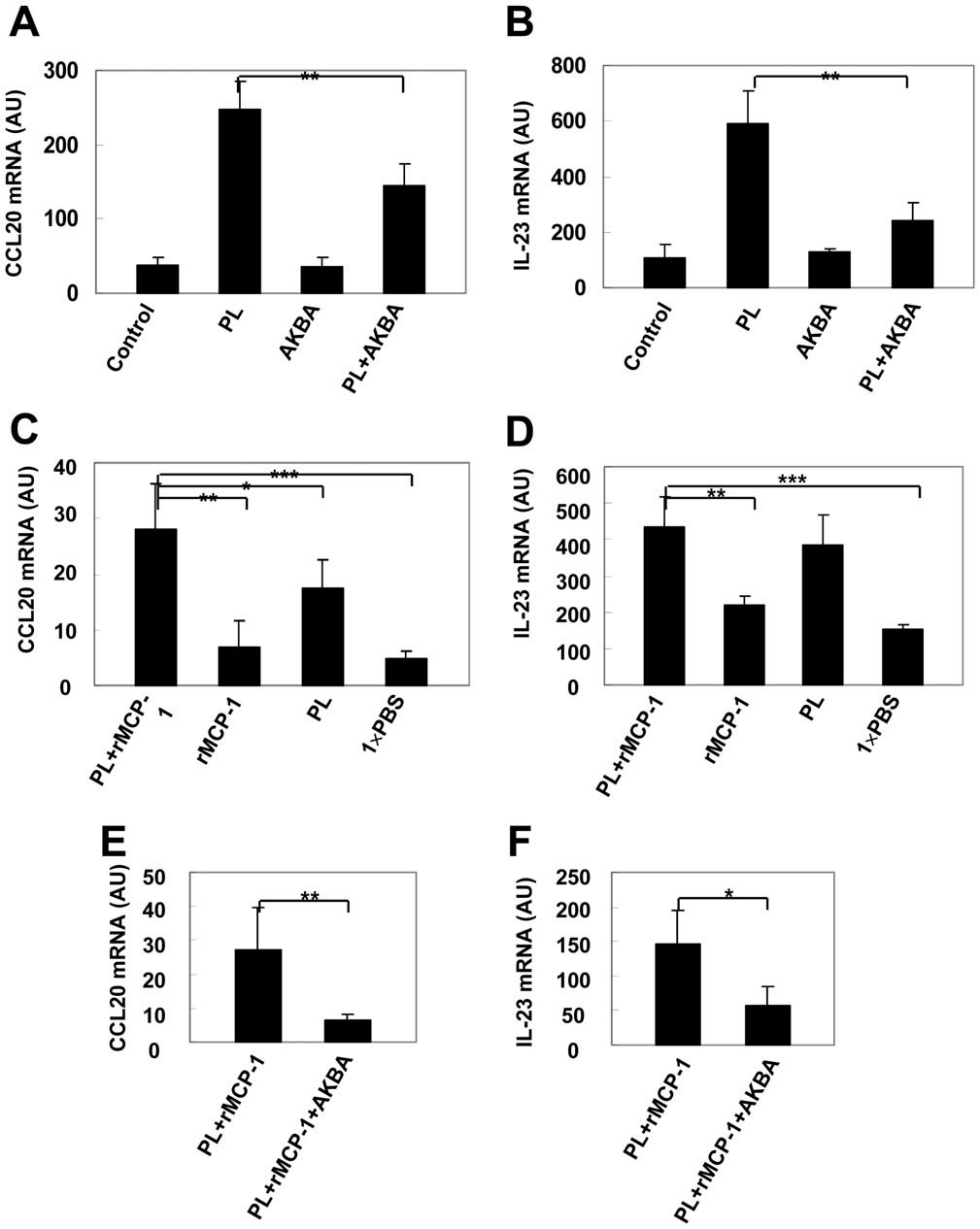
Induction of NF-κB-dependent expression of CCL20 and IL-23 by PL in vitro and in vivo. (**A, B**) Real-time PCR for CCL20 and IL-23 in RNA samples prepared from macrophages with indicated treatment. Ears of WT C57BL/6J mice were injected intradermally with 1.5 µg PL and 30 ng rMCP-1, 30 ng rMCP-1, 1.5 µg PL and 30 ng rMCP-1 and 10 mol/L AKBA or 30 µl 1×PBS alone. (**C, D**) Six hours after injection of PL and rMCP-1, rMCP-1 or PL alone, real-time PCR for CCL20 and IL-23 was performed. (**E, F**) Six hours after injection of PL and rMCP-1 or PL and rMCP-1 and AKBA, real-time PCR for CCL20 and IL-23 was carried out. * *p*<0.05, ** *p*<0.01, *** *p*<0.001, Student's *t* test.

Genes involved in regulating the NF-κB signaling pathway have been suggested to be genetically associated with psoriasis [20]. To investigate a potential role of NF-κB in PL-induced CCL20 and IL-23 expression, we pretreated mouse macrophages with an inhibitor of NF-κB (acetyl-11-keto-b-boswellic acid [AKBA]) before stimulation with PL. We extracted mRNA 6 hours after PL (0.43 CTA U/mL) stimulation for CCL20 and IL-23, and determined the cytokine release. Pretreatment of macrophages with 10 µmol/mL AKBA for 30 minutes inhibited the PL-induced CCL20 and IL-23 release *in vitro*, respectively (**Figure 5A, B**). To further evaluate the role of NF-κB in PL-induced CCL20 and IL-23 *in vivo*, we next injected PL and rMCP-1 with or without AKBA in ears of mice. Real-time PCR was carried out 6 hours after the treatment. Consistently, inhibition of NF-κB resulted in significant decrease of CCL20 production (**Figure 5C, E**) as well as IL-23 production *in vivo* (**Figure 5D, F**). Together with our recent finding [21], our data stressed a pivotal role of the NF-κB signaling pathway in the PL-mediated induction of CCL20 and IL-23, and suggest that NF-κB signaling is critical for the induction of CCL20 and IL-23 in psoriasis.

## Discussion

Because increased levels of PLG activators were found in psoriatic lesions [4], [5], [6], [7], we performed several experiments to examine whether PL, a major serine protease that can be converted from PLG by PLG activators, may be directly involved in pathogenesis of psoriasis. Our major finding is that 1) Diminished PLG resulted in the enhanced amount and activity of PL in psoriasis; 2) PL is a potent cell activator, and is capable of inducing a psoriasiform skin disease that mimics human psoriasis with acanthosis, parakeratosis, subcorneal microabscesses, and dilated lymphatic vessels in mice.

PLG has a critical role in inflammatory responses, and this participation is likely to depend on its interaction with specific receptors on the cell surface [25]. In patients with psoriasis, we detected a diminished expression of PLG and increased production and activity of PL in PBMC or psoriatic lesions, thus confirming an enhanced PLG activity. When the native circulating form of PLG is bound to the cell surface, the generation of PL by PLG activators is markedly stimulated compared to the reaction in solution [26]. However, in agreement with previous findings [4], [5], [6], [7] an increase in the expression and activity of uPA and tPA was observed in psoriatic lesions. Most importantly, we have demonstrated for the first time that patients with psoriasis had 3.43-fold greater PL concentrations (*P*<0.0034) and 2.5-fold greater PL activity (*P* = 0.0034) than controls.

α2-antiplasmin is an important physiologic inhibitor of PL and rapidly inactivates PL by forming a stable PL-inhibitor complex [27]. Herein, we observed that the expression of α2-antiplasmin was markedly decreased in psoriatic lesions compared with controls. This may lead to a situation in which the amount of PL generated exceeds the capacity of α2-antiplasmin to neutralize PL and causes strong immune responses.

Annexin II is a cell-surface PLG receptor which is a calcium-regulated, phospholipid-binding protein on endothelial cells, macrophages, tumor cells and keratinocytes [28], [29]. Consistent with increased PL activity, we have detected pronounced expression levels of annexin II on both the cell surface of PBMC and in lesional skin from patients with psoriasis. Because production of PL was blocked by antibodies against annexin II, over-expression of annexin II may lead to accelerated PL production by its activators [24]. Interestingly, S100A4 which belongs to the S100 family of Ca2^+^-binding proteins, either alone or in a complex with annexin II, accelerates the conversion of PLG to PL [30]. However, a recent study has demonstrated that a strong up-regulation and release of S100A4 was observed in the upper dermis of psoriatic skin, and blocking antibodies against S100A4 resulted in a significant reduction of psoriatic inflammation in a human psoriasis xenograft SCID mouse model [31]. These published data suggest that PL is involved in pathogenesis of psoriasis.

In addition to its fibrinolytic function, PL is a potent cell activator [8]. To directly address whether over-expression of PL is essential for amplification of inflammation in psoriasis, we injected PL together with or without rMCP-1 into mouse ears. We demonstrated that injection of PL or PL and rMCP-1 resulted in enhanced production of psoriasis-related cytokines and subsequent ear skin inflammation with several features resembling human psoriasis. Thus, we conclude that increased PL activity in psoriasis contributes to disease development.

The critical role of CCR6 in psoriasiform inflammation was well documented by the observation of expression of CCR6 and CCL20 in psoriatic lesions [13] and by the finding that both IL-17A- and IL-22-producing cells isolated from lesional skin of patients with psoriasis displayed CCR6 [32]. We have found that injection of PL and rMCP-1 is capable of triggering production of CCL20 and IL-23 together with slight ear skin inflammation in CCR6−/− mice, suggesting that macrophages, DCs or keratinocytes, rather than CCR6^+^ T cells, are the major source of CCL20 and IL-23 under stimulation of PL *in vivo*. However, production of IL-22, IL17A and IFN-γ was severely decreased in CCR6−/− ears treated with PL and rMCP-1. Furthermore, we demonstrated that no development of skin inflammation was observed in SCID mice treated with PL and rMCP-1. Thus, our data clearly indicate that induction of psoriasiform skin changes by PL requires CCR6^+^ T cells.

A genome-wide scan has revealed that the NF-κB signaling pathway has a critical role in development of psoriasis [20]. Here we investigated whether the NF-κB signaling pathway is involved in PL-induced pro-inflammatory gene expression *in vitro* and *in vivo*. Using a natural NF-κB inhibitor, AKBA [21], we demonstrated that in the presence of AKBA, the expression levels of CCL20 and IL-23 stimulated by PL were severely decreased, confirming that PL-induced signal transduction entails the activation of the transcription factor NF-κB. In summary, psoriasis patients have elevated levels of PLG activators which lead to a marked increase in the conversion of cell-bound PLG to the active PL. The excessive amount of PL in psoriasis patients may not be neutralized by α2-antiplasmin because this inhibitor is significantly lower than in normal controls. Under inflammatory conditions, PL directly binds to its cell surface receptor, annexin II, which is expressed on activated monocytes/macrophages, DCs and keratinocytes, a critical initiating step in the pathogenesis of psoriasis in predisposed individuals. Activation of these inflammatory cells induce pro-inflammatory gene expression, including expression of CCL20, IL-23, TNF-α and others, which contribute to the recruitment/activation of CCR6^+^ pathogenic T cells. These T cells produce large amounts of IFN-γ, IL-17 and IL-22, leading to the skin changes observed in human psoriasis (**Figure S1**). Collectively, our findings establish PL derived from the conversion of PLG by PLG activators in inflammatory conditions as an essential element that may initiate and/or worsen psoriasis by inducing the recruitment and activation of skin-homing pathogenic T cells or by directly activating keratinocytes in patients. We believe that targeting PL receptor - annexin II - has therapeutic potential for the treatment of psoriasis.

## Materials and Methods

### Ethics statement

Mice were kept under specific pathogen-free (SPF) conditions in compliance with the National Institutes of Health *Guide for the Care and Use of Laboratory Animals* with the approval (SYXK-2003-0026) of the Scientific Investigation Board of Shanghai Jiao Tong University School of Medicine, Shanghai, China. To ameliorate any suffering of mice observed throughout these experimental studies, mice were euthanized by CO_2_ inhalation.

### Mice

WT C57BL/6J mice, SCID mice, WT BALB/cByJ mice, and CCR6−/− (B6.129P2-CCR6^tm1Dgen^/J) mice were purchased from the Jackson Laboratory (Bar Harbor, ME).

### Clinical samples

Clinical samples were obtained following protocols approved by Shanghai Jiao Tong University School of Medicine Review Board and following the Declaration of Helsinki Principles. Psoriatic lesional biopsies or PBMC from patients with moderate-to-severe chronic plaque psoriasis were collected simultaneously (*n* = 12). Sex and age-matched control samples were from normal volunteers (*n* = 15). Patients treated with immunosuppressive therapies were excluded.

### Flow cytometry analysis

PBMCs from either patients with psoriasis or controls were prepared as previously published [33]. The following antibodies were used for flow cytometry, all in conjunction with AlexaFluor 555 (Caltag Laboratories): anti-human PLG (Santa Cruz), anti-human uPA, anti-human uPA (R&D), and anti-human annexin A2 (ABGENT). Isotype IgGs were used as controls for all experiments.

### Immunofluorescent staining

Frozen cryosections of skin from patients and volunteers or mice with the indicated genotype or treatment were fixed in ice-cold acetone for 10 min before staining. The following antibodies were used for immunofluorescent staining, all in conjunction with AlexaFluor 555 or AlexaFluor 488 (Caltag Laboratories): anti-human PLG (Santa Cruz Biotechnology), anti-human uPA (Abcam), anti-human tPA (Abcam), anti-human annexin A2 (Abgent), anti-mouse CD11b (Biolegend), and anti-mouse TNF-α (Biolegend). 4′,6-Diamidino-2-phenylindole (DAPI) (Fluka) was used for the staining of nuclei. Anti-mouse IgG2b, K (BioLegend, Cat. No.: 400302), anti-mouse IgG1, K (BioLegend, Cat. No.: 400102) and anti-Rat IgG2b, K (BioLegend, Cat. No.: 400602) were used as control stainings.

Antibodies were diluted in antibody diluent (Dako). Immunostained samples were analyzed with a fluorescence microscope (Zeiss Axio Scope A1). Isotype IgGs were used as control stainings.

### ELISA

Human total PL was measured using ELISA kits for plasmin-alpha-2-antiplasmin (PAP) complex (Technoclone GmbH). Plasma from patients with chronic plaque-type psoriasis and healthy volunteers were collected and frozen immediately until analysis. The assay was performed following the manufacturer's protocol.

### Chromozym PL activity assay

Chromozym PL (Roche) was used to measure the activity of PL. Equivalent amounts of plasma protein (5 µg) from psoriasis patients and controls were diluted according the manufacturer's protocol. Triplicates of each sample were dispensed into 96-well plates and incubated at 37°C. After incubation, the absorbance was read at 405 nm.

### Cell culture

Purified human PL was purchased from Athens Research & Technology. It was free of lipopolysaccharide contamination as analyzed by the Pyrogent LAK assay (Lozna). PL activity is given in Committee on Thrombolytic Agents (CTA) units/mL. The murine macrophage cell line RAW264.7 was cultured in RPMI 1640 (Invitrogen, Carlsbad, CA) supplemented with 10% FCS. Macrophages were treated with 10 µg/mL AKBA for 30 min and subsequently stimulated with PL in lysine-free RPMI 1640 (Sigma) for six hours before harvesting for RNA extraction.

### Quantitative RT-PCR

RNA extraction and PCR were carried out as previously described [15]. Primer sequences used were designed for murine IFN-γ (5′-ATCTGGAGGAACTGGCAAAA-3′, 5′-TGACGCTTATGTTGTTGCTG-3′), TNF-α (5′-GAACTGGCAGAAGAGGCACT-3′, 5′-AGGGTCTGGGCCATAGAACT-3′), IL-17A (5′-GCTCCAGAAGGCCCTCAGA-3, 5′-CTTTCCCTCCGCATTGACA-3′, CCL20 (5′-GACTGTTGCCTCTCGT-3′, 5′-TGACTCTTAGGCTGAGGA-3′), IL-23 (5′-CAGCAGCTCTCTCGGAATCT-3′, 5′-TGGATACGGGGCACATTATT-3′), IL-6 (5′-CACAAGTCCGGAGAGGAGAC-3′, 5′-CAGAATTGCCATTGCACAAC-3′), IL-1α (5′-GCAACGGGAAGATTCTGAAG-3′, 5′-TGACAAACTTCTGCCTGACG-3′), IL-22 (5′-GTCAACCGCACCTTTATGCT-3′, 5′-GTTGAGCACCTGCTTCATCA-3′), and β-actin (5′-TGGAATCCTGTGGCATCCATGAAAC-3′, 5′-TAAAACGCAGCTCAGTAACAGTCCG-3′). Cytokine and β-actin levels were calculated relative to the amounts found in a standard sample, and murine β-actin was employed for normalization as previously reported [15].

### Western blot analysis

Lysates of psoriatic skin of patients or normal skin of healthy individuals were prepared as previously described [21]. Anti-human PLG (R&D, Cat. No.: MAB1939), anti-human annexin II (Abcam®, Cat. No.:ab54771), and anti-human α2-antiplasmin (Abcam®, Cat. No.:ab30994) were used for western blotting. Western blotting was performed as previously published [8].

### PL-induced skin inflammation model

The ears of mice with the indicated genotypes were injected intradermally every other day for different time periods with 30 µl 1×PBS alone or containing either 1.5 µg PL and/or 30 ng recombinant murine MCP-1 (R&D). Injected ear samples were frozen directly in liquid nitrogen for mRNA extraction, fixed in 10% neutral buffered formalin for histology, or embedded and frozen in OCT for immunofluorescent staining.

### Statistical analysis

Quantitative data are presented as mean values ± standard deviation (SD). Statistical significance was determined by the two-tailed Student's t test or the Mann-Whitney U test in cases of a non-Gaussian distribution. Differences were considered to be statistically significant at values of *p*<0.05 *, *p*<0.01 **, *p*<0.001 ***. Statistical analysis was performed with GraphPad Prism 5.01 software.

## Supporting Information

Figure S1 **A model for how plasmin is involved in psoriasis pathogenesis.** Psoriasis patients have elevated plasminogen activators which lead to a marked increase in conversion of cell-bound plasminogen to the active plasmin. Insufficient production of α2-antiplasmin results in enhanced activity of plasmin in psoriasis patients. Binding of plasmin to its cell surface receptor annexin II activates monocytes / macrophages, DCs and keratinocytes, a critical initiating step in the pathogenesis of psoriasis in predisposed individuals. Activation of these inflammatory cells induce pro-inflammatory gene expression including CCL20, IL-23 and TNF-α that contribute to the recruitment / activation of CCR6^+^ pathogenic T cells, which produce large amount of IFN-γ, IL-17 and IL-22, amplifying inflammation in psoriasis. Mφ, macrophages; KC, keratinocytes; DC, dendritic cells.
(TIF)
