# Supplementary figures and images for: Plasmin Plays an Essential Role in Amplification of Psoriasiform Skin Inflammation in Mice
Source: PLoS One. 2011 Feb 2;6(2):e16483. doi: 10.1371/journal.pone.0016483 (PMC3032787; doi:10.1371/journal.pone.0016483)

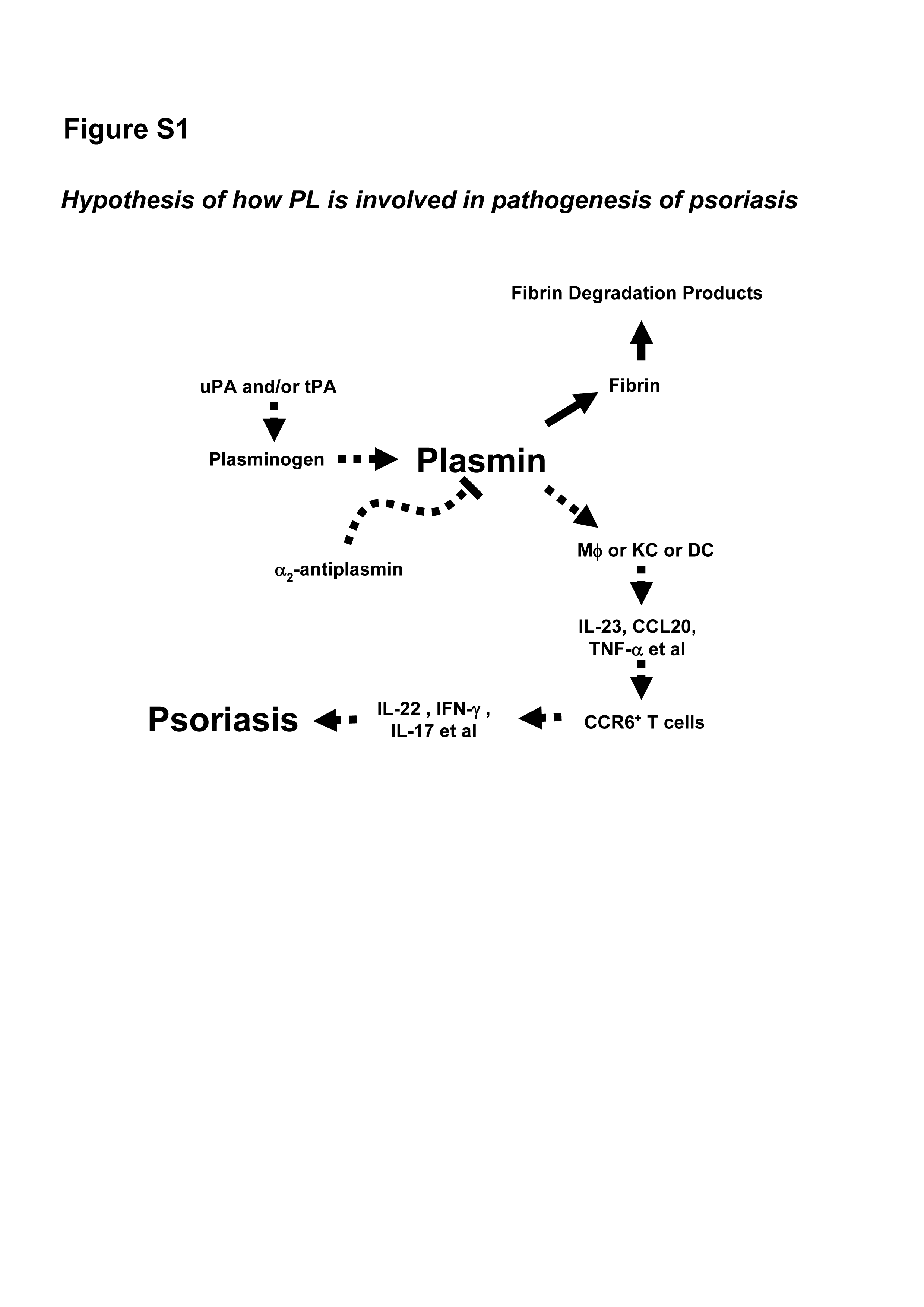

Supplement: Figure S1 — A model for how plasmin is involved in psoriasis pathogenesis. Psoriasis patients have elevated plasminogen activators which lead to a marked increase in conversion of cell-bound plasminogen to the active plasmin. Insufficient production of α2-antiplasmin results in enhanced activity of plasmin in psoriasis patients. Binding of plasmin to its cell surface receptor annexin II activates monocytes / macrophages, DCs and keratinocytes, a critical initiating step in the pathogenesis of psoriasis in predisposed individuals. Activation of these inflammatory cells induce pro-inflammatory gene expression including CCL20, IL-23 and TNF-α that contribute to the recruitment / activation of CCR6+ pathogenic T cells, which produce large amount of IFN-γ, IL-17 and IL-22, amplifying inflammation in psoriasis. Mφ, macrophages; KC, keratinocytes; DC, dendritic cells. (TIF) [file pone.0016483.s001.tif]
